# Arachidonic Acid Metabolism Pathway Is Not Only Dominant in Metabolic Modulation but Associated With Phenotypic Variation After Acute Hypoxia Exposure

**DOI:** 10.3389/fphys.2018.00236

**Published:** 2018-03-16

**Authors:** Chang Liu, Bao Liu, Lu Liu, Er-Long Zhang, Bind-da Sun, Gang Xu, Jian Chen, Yu-qi Gao

**Affiliations:** ^1^Institute of Medicine and Hygienic Equipment for High Altitude Region, College of High Altitude Military Medicine, Army Medical University, Third Military Medical University, Chongqing, China; ^2^Key Laboratory of High Altitude Environmental Medicine, Army Medical University, Third Military Medical University, Ministry of Education, Chongqing, China; ^3^Key Laboratory of High Altitude Medicine, People's Liberation Army, Chongqing, China; ^4^The 12th Hospital of Chinese People's Liberation Army, Kashi, China

**Keywords:** metabolomics, transcriptomics, WGCNA, hypoxia, arachidonic acid

## Abstract

**Background:** The modulation of arachidonic acid (AA) metabolism pathway is identified in metabolic alterations after hypoxia exposure, but its biological function is controversial. We aimed at integrating plasma metabolomic and transcriptomic approaches to systematically explore the roles of the AA metabolism pathway in response to acute hypoxia using an acute mountain sickness (AMS) model.

**Methods:** Blood samples were obtained from 53 enrolled subjects before and after exposure to high altitude. Ultra-performance liquid chromatography-quadrupole time-of-flight mass spectrometry and RNA sequencing were separately performed for metabolomic and transcriptomic profiling, respectively. Influential modules comprising essential metabolites and genes were identified by weighted gene co-expression network analysis (WGCNA) after integrating metabolic information with phenotypic and transcriptomic datasets, respectively.

**Results:** Enrolled subjects exhibited diverse response manners to hypoxia. Combined with obviously altered heart rate, oxygen saturation, hemoglobin, and Lake Louise Score (LLS), metabolomic profiling detected that 36 metabolites were highly related to clinical features in hypoxia responses, out of which 27 were upregulated and nine were downregulated, and could be mapped to AA metabolism pathway significantly. Integrated analysis of metabolomic and transcriptomic data revealed that these dominant molecules showed remarkable association with genes in gas transport incapacitation and disorders of hemoglobin metabolism pathways, such as ALAS2, HEMGN. After detailed description of AA metabolism pathway, we found that the molecules of 15-d-PGJ2, PGA2, PGE2, 12-O-3-OH-LTB4, LTD4, LTE4 were significantly up-regulated after hypoxia stimuli, and increased in those with poor response manner to hypoxia particularly. Further analysis in another cohort showed that genes in AA metabolism pathway such as PTGES, PTGS1, GGT1, TBAS1 et al. were excessively elevated in subjects in maladaptation to hypoxia.

**Conclusion:** This is the first study to construct the map of AA metabolism pathway in response to hypoxia and reveal the crosstalk between phenotypic variation under hypoxia and the AA metabolism pathway. These findings may improve our understanding of the advanced pathophysiological mechanisms in acute hypoxic diseases and provide new insights into critical roles of the AA metabolism pathway in the development and prevention of these diseases.

## Introduction

Hypoxia is a common phenomenon associated with multiple pathophysiological processes, including inflammation, necrosis, and apoptosis, and observed in various clinic conditions such as myocardial ischemia and reperfusion as well as stroke (Michiels, 2004). The arachidonic acid (AA) metabolism pathway was shown to exhibit remarkable metabolic alterations after hypoxia exposure, but its role is controversial (Liao et al., 2016). As a part of the innate immune response, AA and its metabolites are known to induce or suppress inflammation (Bennett and Gilroy, 2016). In addition, the products of the AA cascade are shown to display multidirectional and often conflicting roles in endothelial and nerve cells, such as inhibition or promotion of cell proliferation and aggravation or alleviation of oxidative stress, in response to stimuli (Bogatcheva et al., 2005; Rink and Khanna, 2011). Although the partial function of this pathway was identified, the mechanism underlying the expression of the AA metabolism pathway and its role under acute hypoxic conditions are questionable.

Acute hypobaric hypoxia, one of the most common hypoxia types, occurs in response to the exposure to high altitude and initiates systematic pathophysiological alterations. Acute hypobaric hypoxia may serve as a principal etiological factor for acute mountain sickness (AMS) (Sun et al., 2017). Therefore, AMS is considered as a potent channel and an *in vivo* model to improve our understanding of the various acute hypoxic diseases with high-quality samples from self-controlled studies (Subudhi et al., 2010). In addition, mal-adaptation to hypoxia is characterized by symptoms such as headache, nausea, and vomiting that may progress into high altitude pulmonary edema or high altitude cerebral edema-the leading lethal diseases for mountaineers (Johnson and Luks, 2016). With ~15 million people traveling to areas of high altitude (>2,500 m) annually in China alone (Gonggalanzi et al., 2016), it is important to unravel the mechanisms underlying AMS.

The technical platforms of system biology allow researchers to explore systematic alterations under hypoxia exposure. Metabolomic studies have demonstrated that metabolic reprogramming with dramatic turbulence in various metabolic pathways, including linoleic acid metabolism, purinergic signaling, and glycolysis, was implied in response to acute hypoxia (D'Alessandro et al., 2016; Liao et al., 2016). In addition, a recent study on acute hypoxia stimuli based on RNA-seq approach indicated that the disturbance in the inflammation response presented by the reduction in interleukin (IL)-10 was highly related to AMS incidences (Liu et al., 2017a). However, studies based on a single set of omics technology failed to extensively explain the alterations occurring at different levels during physiologic or pathophysiological responses, such as disabilities in interpreting metabolic regulatory processes in metabolomics (Kan et al., 2017). The development of efficient analysis methods and algorithms allows better insights, beyond those available from individual strategies through the integration of diverse omics (Li et al., 2016; Sun and Hu, 2016). In particular, the method of weighted gene co-expression network analysis (WGCNA), which could exhibit multiple network-level interactions, is effective for the identification of gene-to-metabolite relations and construction of the underlying complex regulatory networks (Acharjee et al., 2016; Liu et al., 2017b).

Here, we aimed to integrate clinical features, metabolomic datasets, and transcriptome profiling for the construction of the co-expression network to provide a better understanding of the AA metabolism pathway after acute hypoxia exposure. Our results identified that the AA metabolism pathway, a highly regulated metabolic pathway, was essential in response to acute hypoxia. Furthermore, the AA metabolism pathway may account for variations in response patterns after acute hypoxia stimuli. Our results may improve the understanding of AMS and provide new clues to counteract the effects of hypoxic diseases.

## Materials and methods

### Enrolled subjects and experimental procedures

We enrolled 53 male subjects with intact clinical phenotype data of heart rate (HR), oxygen saturation (SpO_2_), Lake Louise score (LLS), systolic blood pressure (SBP), diastolic blood pressure (DBP), and hemoglobin (HB) before and after high altitude exposure (5,300 m). These subjects were covered in our previous study (Liao et al., 2016). Physical and physiological characteristics [mean ± standard deviation (SD)] for enrolled subjects were as follows: age, 21.6 ± 2.0 years; height, 172 ± 1 cm; and body weight, 64.9 ± 2.3 kg. Exclusion criteria included prior exposure to high altitude, presence of cardiovascular pathologies or other diseases, and history of drug prescribed for high altitude. These volunteers traveled to an area of 5,300 m from the starting point of 1,400 m within 4 days, as illustrated in our previous article (Liao et al., 2016). Their clinical symptoms of HR, SpO_2_, LLS, SBP, DBP, HB were monitored before and after hypoxia stimuli. The local ethics committee of the Third Military Medical University approved the experimental design, methods of clinical data collection, and strategies of data evaluation. These procedures were performed in accordance with the approved guidelines. Written informed consents were obtained from all participants.

### Metabolomics analysis

Metabolic profiling was performed, and metabolic data were obtained as previously described (Liao et al., 2016). Briefly, blood was drawn from a peripheral vein in the morning soon after waking before ascending to high altitudes and after 3 days high-altitude exposures. Blood samples were centrifuged to separate serum at 14,000 × g for 15 min at 4°C. Then, these plasma samples were delivered to Chongqing for further metabolomics analysis in the courier filed with dry ice.

Metabolomics analysis was conducted on an Agilent 1290 Infinity LC system (Agilent, Santa Clara, CA, USA). Chromatographic separations were performed on an ACQUITY UHPLC HSS T3 C18 column (2.1 × 100 mm, 1.8 μm; Waters, Milford, Ireland) at 45°C. The metabolomics detection was performed totally in accordance to the manufacturers' protocols of all devices. Raw LC-MS data were converted to mzData formats via Agilent MassHunter Qualitative software (Agilent, Santa Clara, CA, USA). The program XCMS (version 1.40.0) (https://xcmsonline.scripps.edu/) was used to preprocess the raw data, including peak detection, peak matching, matched filtration, and nonlinear alignment of data, with the default parameters (and snthresh, 5; bw, 10; fwhm, 10).

The resulting matrix from 53 subjects comprising retention time, mass-to-charge ratio (m/z), and normalized ion intensities was introduced into the subsequent analysis based on R platform (https://www.r-project.org). The model of partial squares discriminant analysis (OPLS-DA) was carried out to separate pre- and post-hypoxia in metabolic profiling. Variable importance project (VIP) was used to reveal discriminatory metabolites which were enrolled in the classification effects of hypoxia was calculated. In addition, adjusted *p*-value from the paired *t*-test and fold-change value (FC) were executed to discovery dominant metabolites. Pathway analysis was established by MetaboAnalyst platform (http://www.metaboanalyst.ca).

### Transcriptomic profiling detection

To improve the reliability of the integrated analysis to uncover interactions at metabolic and transcriptional level, 11 individuals were randomly selected and subjected to metabolomic and RNA-seq detection (Li et al., 2016). RNA sequencing was performed as previously described (Liu et al., 2017a) with minor modifications. Briefly, after extraction, total RNA was quantified by spectrophotometer (NanoDrop 200, Thermo Scientific, Delaware, USA). mRNA-seq libraries were constructed according to the TruSeq RNA Sample Prep Kit v2 (Illumina) and sequenced on an Illumina HiSeq 2000 sequencer, following the manufacturer's instructions. after filtered, the clean reads were aligned to human genome hg19 based on HISAT and assembled by Stringtie (Li et al., 2014). The expression value of each transcript was subsequently calculated based on methods of Fragments Per Kilobase of exon model per Million mapped fragments (FPKM). In addition, GSE52209 datasets of blood samples from individuals suffering from adaptation or mal-adaptation to high altitude exposure were downloaded from Gene Expression Omnibus (GEO) datasets. After annotation, limma package was employed to normalize and detect the significantly differential genes involved in diverse response manner to hypoxia (Ritchie et al., 2015). ClusterProfiler package was used to determine enriched Gene Ontology (GO) terms in differentially expressed gene sets (Yu et al., 2012).

### Weighted gene co-expression network analysis (WGCNA)

WGCNA, a robust tool for the integrative network analysis, is widely used in complex network construction (Liu et al., 2017b). According to the WGCNA protocol, we constructed clusters of genes firstly based on the matrix of pairwise correlations calculated across the selected samples. Then, we performed the network construction and module detection based on an appropriate soft-thresholding power. The default minimum cluster merge height of 0.25 was retained. These clusters identified by WGCNA are called modules, which represented a group of highly interconnected genes with similar expression profiles across the enrolled subjects. Further, we determined correlations among expression modules and clinical traits for all subjects. Hub genes were identified based on the connectivity among all nodes. Network was illustrated by Cytoscape software (Smoot et al., 2011).

### Statistical analysis

Differences before and after hypoxia exposure were determined by paired *t*-test. The unpaired Student's *t*-test was used to compare the expression between subjects with diverse response patterns to hypoxia. Statistical analysis was performed by GraphPad software (GraphPad Prism, USA). The data in this study were presented as the mean ± SD. A two-tailed p value < 0.05 was considered as significant, unless specifically indicated.

## Results

### Baseline clinical characteristics of enrolled subjects

**Figure 1:**
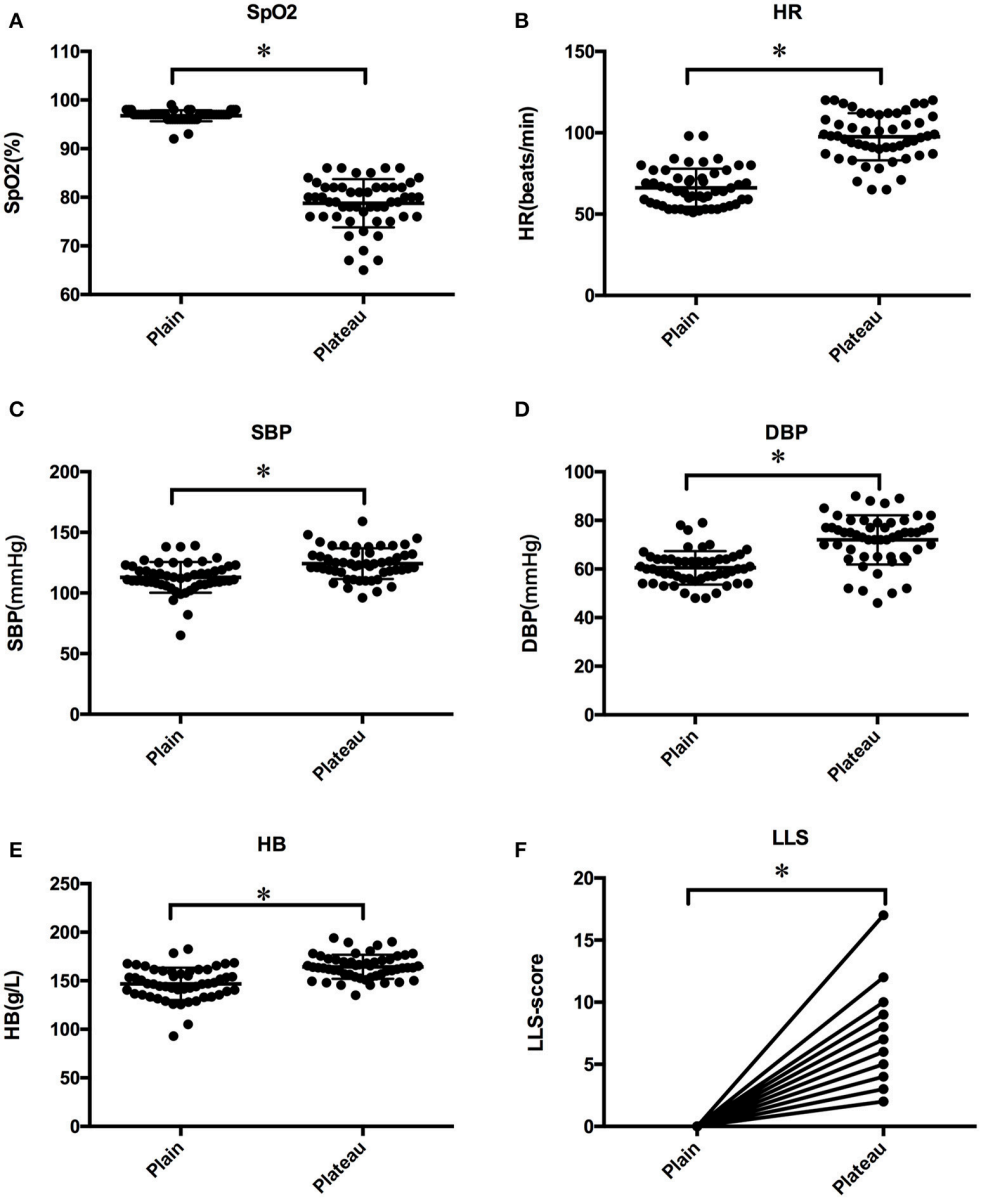
Baseline clinical characteristics of SpO_2_ **(A)**, HR **(B)**, SBP **(C)**, DBP **(D)**, HB **(E)**, and LLS **(F)** for enrolled subjects before and after high altitude exposure. Statistical significance is indicated as *p < 0.05.

The response of subjects to acute hypobaric hypoxia was assessed by various physiological parameters. As shown in Figure 1A, SpO_2_, reflective of the oxygen concentration in the blood, significantly declined under hypoxia environment (*p* < 0.05; Netzer et al., 2017). Among the essential compensatory measurements, HR (Figure 1B), SBP (Figure 1C), DBP (Figure 1D), and HB (Figure 1E) increased along with hypoxia (*p* < 0.05), indicative of the comprehensive response manner to acute hypoxia stimuli. A significant increase in LLS was observed for all individuals (Figure 1F; *p* < 0.05).

### Arachidonic acid metabolism pathway shows a remarkable correlation with phenotypic alterations after hypoxia exposure

**Figure 2:**
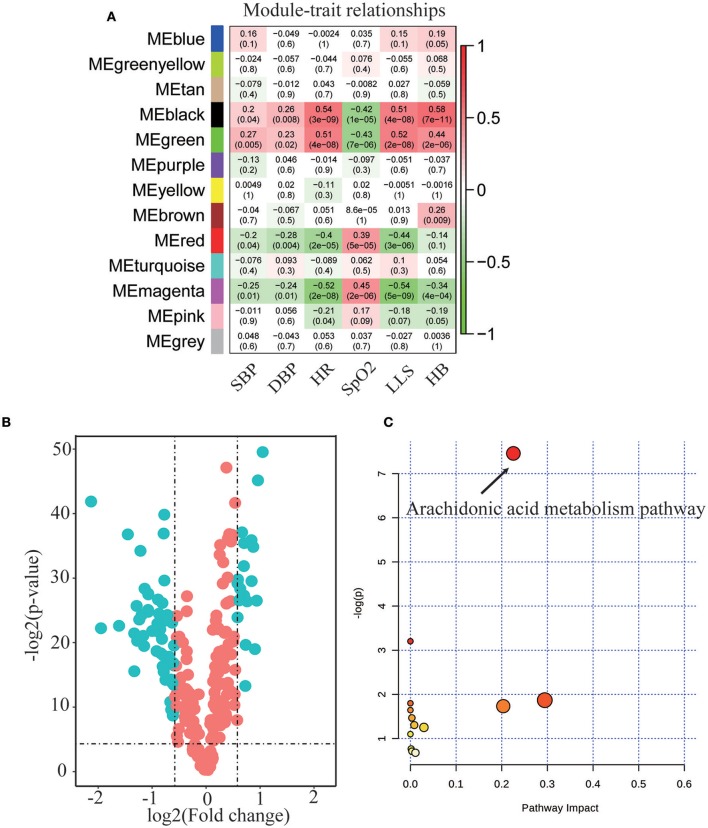
Metabolic profiling analysis in response to acute hypoxia exposure. **(A)** WGCNA analysis identified four connectivity-based modules with high correlation to clinic features of heart rate (HR), oxygen saturation (SpO_2_), Lake Louise score (LLS), systolic blood pressure (SBP), diastolic blood pressure (DBP), and hemoglobin (HB). High correlation values are indicated by red and negative correlations, in green color. **(B)** Comparison of all metabolites from plasma of subjects before and after hypoxia exposure. The volcano plot displays the relationship between fold change and significance using a scatter plot view. The green points in the plot represent the differential metabolites with statistical significance. **(C)** Enrichment pathway analysis for the dominant metabolites identified in the volcano plot before and after hypoxia exposure.

Metabolomic analysis of blood samples from individuals before and after high altitude exposure revealed a total of 1,720 compounds. As indicated in Figure 2A, the method of WGCNA identified that four modules (MEgreen, MEred, MEmagenta, and MEblack) showed significant correlations with all clinical features of SBP, LBP, HR, SpO_2_, LLS, and HB. To further explore influential metabolites in these modules, a volcano plot for these molecules was obtained (Figure 2B). At a cutoff point of *p* < 0.05, FC ≥ 1.5, and VIP > 1.5, a total of 36 metabolites showed significant alteration and comprised nine downregulated molecules and 27 elevated metabolites (Table 1). Moreover, the enrichment analysis demonstrated that AA metabolism pathway was particularly significant in the metabolic alterations in response to acute hypoxia (Figure 2C).

**Table 1:** Summary of the influential metabolites in 53 subjects at high altitude relative to plain.

**Metabolites**	***P*-value**	**Fold change**	**VIP**
12-Oxo-trihydroxy-leukotriene B4	2.04E-07	0.26	2.29
Bilirubin	1.57E-07	0.33	3.26
Undecanoylcarnitine	8.49E-12	0.37	1.75
15(S)-HETE	2.06E-05	0.40	1.85
Oleic acid	1.85E-08	0.41	4.47
L-Hexanoylcarnitine	8.01E-08	0.42	1.84
Ceramide (d18:1/16:0)	4.99E-11	0.43	2.20
15-Deoxy-d-12,14-PGJ2	4.84E-08	0.44	1.60
3, 5-Tetradecadiencarnitine	1.38E-06	0.45	2.33
20-Hydroxy-leukotriene E4	2.89E-09	0.45	1.57
Pimelylcarnitine	3.49E-08	0.48	2.23
3-hydroxydecanoyl carnitine	4.50E-09	0.48	2.25
9-Hexadecenoylcarnitine	5.37E-09	0.48	2.07
Dodecanoylcarnitine	2.75E-07	0.50	2.15
Decanoylcarnitine	2.35E-06	0.53	2.60
Tetradecanoylcarnitine	1.23E-07	0.54	1.73
trans-2-Dodecenoylcarnitine	2.80E-07	0.54	2.28
Linoleic acid	4.28E-08	0.55	4.33
DG(15:0/18:0/0:0)	2.29E-07	0.55	1.90
LysoPE(14:0/0:0)	1.35E-08	0.57	2.98
Palmitic acid	1.23E-05	0.57	2.87
N-methylphenylalanine	7.75E-12	0.58	1.55
Tiglylglycine	1.02E-12	0.58	2.93
21-Hydroxypregnenolone	8.95E-08	0.59	1.54
L-Octanoylcarnitine	5.25E-05	0.59	2.00
Arachidonic acid	1.17E-07	0.65	2.61
Alpha-Linolenic acid	8.90E-05	0.66	1.55
LysoPC(20:2)	6.82E-12	1.59	3.08
LysoPC(P-18:0)	2.57E-10	1.62	2.14
LysoPC(20:0)	6.09E-09	1.63	1.79
LysoPE(0:0/22:0)	2.17E-11	1.63	2.26
Stearoylcarnitine	1.22E-06	1.66	2.26
Glycocholic acid	1.14E-08	1.69	2.62
Deoxyinosine	3.28E-11	1.83	1.74
Deoxyribose 1-phosphate	1.92E-06	1.87	1.69
LysoPC(18:2(9Z,12Z))	1.23E-15	2.07	2.346

### Integrative network-based analysis revealed mechanisms of AA metabolism pathway

**Figure 3:**
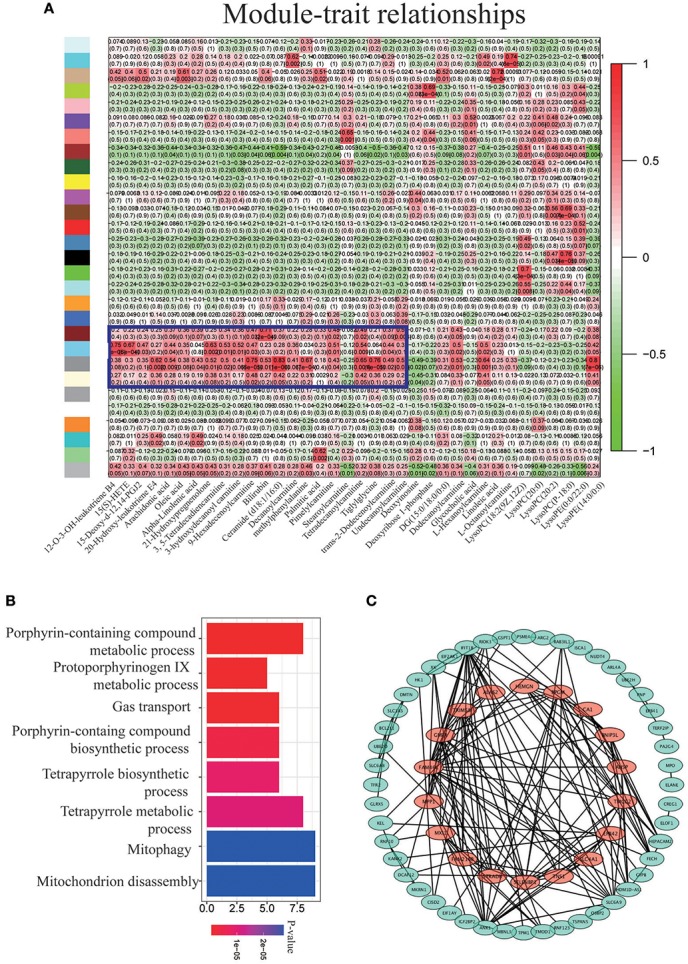
Integrated analysis of metabolomic and transcriptomic profiling. **(A)** Unsupervised hierarchical cluster of correlation coefficients (kME and normalized metabolite values). High correlations are colored in red and low correlations, in green. **(B)** GO analysis for the modules inside the violet frame. **(C)** Network visualization of genes with high correlations to the AA metabolism pathway.

**Figure 4:**
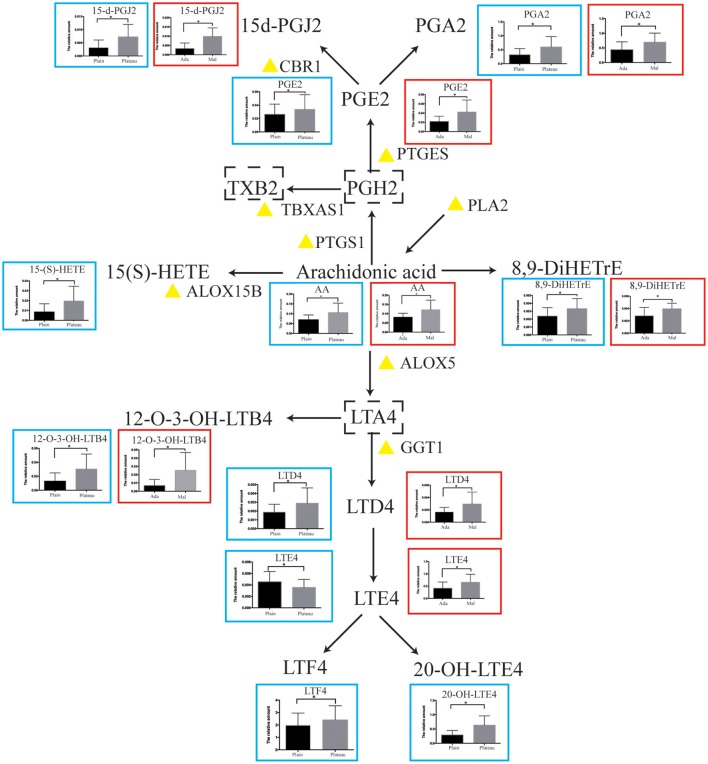
Schematic overview of the AA metabolism pathway. The alterations in metabolites are shown in a blue frame (plain VS plateau), while the changes between adaptation(Ada) subjects and maladaptation(Mal) individuals to hypoxia are presented in a red box. The yellow triangle indicates the key enzymes validated in another cohort. The metabolites labeled by a dotted box represent no detection. Statistical significance is indicated as *p < 0.05.

The physiological characteristics corresponding to SpO_2_, HR, HB, LLS, SBP, and LBP for subjects evaluated by metabolomics and transcriptomics are listed in Supplemental Figure 1. These subjects chose for both omics detection, posed clinical features similar to those of the whole population after hypoxia stimuli. To provide a comprehensive interaction network between metabolomic and transcriptomic data sets, WGCNA was performed to identify connectivity-based modules comprising genes closely related to the aforementioned impact metabolites. As illustrated in Figure 3A, gene modules labeled by a violet frame were significantly correlated with these molecules, especially to metabolites of the AA metabolism pathway. As shown in Figure 3B, heme catabolism, gas transport, and porphyrin-related metabolism pathways were dominant in these gene modules after GO analysis (*p* < 0.05). To further clarify the mechanisms underlying the AA metabolism pathway after hypoxia exposure, genes with the highest connectivity from the co-expression network and a correlation coefficient >0.65 to at least one molecule among 13 metabolites in the AA metabolism pathway are circled in red in Figure 3C. These marked genes promoted proliferation and regulation of red blood cell metabolism. The detailed information is listed in Figure 4.

### Detailed AA metabolism pathway along with hypoxia stimuli

A map of metabolites in AA metabolism pathway was constructed in Figure 4. Alterations in these molecules before and after hypoxia exposure are listed in the blue box next to each metabolite. To elucidate the differences in subjects exhibiting diverse response patterns to hypoxia, these individuals were divided into two groups based on their LLS scores. Individuals with an LLS score lower than 4 were enrolled in the control group, while those with an LLS score higher than 9 were included in the group of mal-adaptation to hypoxia. Clinical characteristics of each group are listed in Supplemental Table 1. The metabolites exhibiting significant differences between control and mal-adjustment group are shown in the red box in Figure 4. As observed, the AA metabolism pathway was upregulated after hypoxia exposure and further elevated in those with poor response to hypoxia.

### Validation of AA metabolism pathway in another dataset

**Figure 5:**
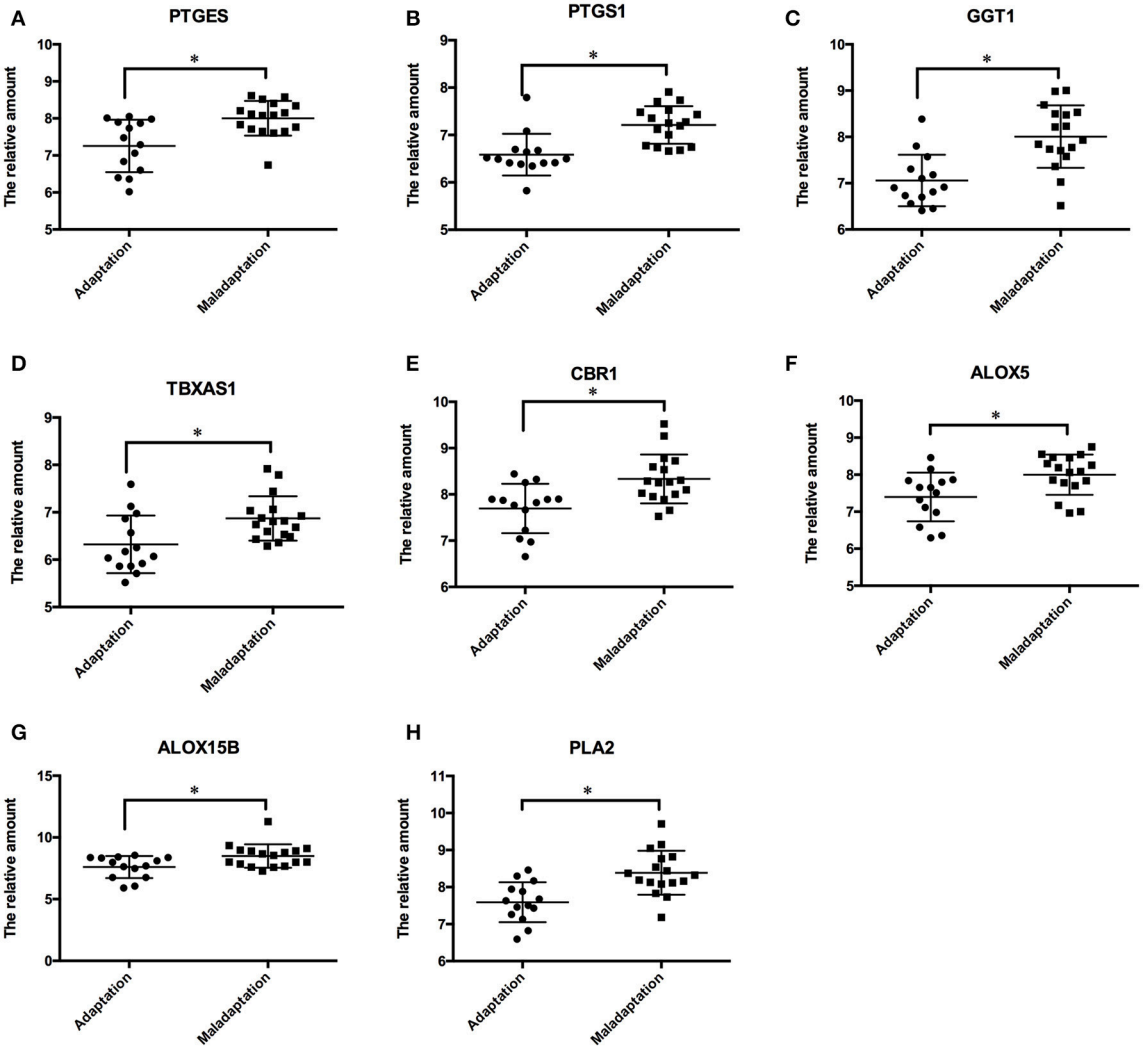
Validation of the AA metabolism pathway in another cohort. The genes of PTGES **(A)**, PTGS1 **(B)**, GGT1 **(C)**, TBXAS1 **(D)**, CBR1 **(E)**, ALOX5 **(F)**, ALOX15B **(G)**, PLA2 **(H)** were significantly up-regulated in those who exhibited mal-adaptation to high altitude exposure. Statistical significance is indicated as *p < 0.05.

We validated our findings in another population of GSE52209 from GEO database that comprised 14 subjects as the control group and 17 individuals suffering from mal-adaptation to high altitude. In comparison to individuals that adapted to hypoxia, those with poor response to acute hypoxia showed a significant upregulation (*p* < 0.05, Figure 5) in the expression of genes such as *GGT1, PTGES, PTGS1, CBR1*, and *ALOX12* encoding key enzymes of the AA metabolism pathway. The effects of these genes on AA metabolism pathways are labeled with a yellow triangle in Figure 4.

## Discussion

Here, we performed an integrated analysis of metabolomic and transcriptomic expression profiling with the whole blood of individuals that experienced acute hypoxia. These approaches revealed pivotal roles of the AA metabolism pathway in the phenomenon of metabolic reprogramming during acute hypoxia exposure. Co-expression network analysis further identified that the AA metabolism pathway showed a significant positive correlation with transcriptional alterations, particularly with genes involved in porphyrin metabolism and gas transport pathways. Taken together, this is the first study to provide systematic changes in the AA metabolism pathway and reveal its potential mechanisms in response to hypoxia exposure.

We found an increase in the expression of molecules such as AA, prostaglandin A2 (PGA2), and cysteinyl leukotrienes (Cys–LTs) in AA metabolism pathway after hypoxia exposure. These molecules were important sources of pro-inflammatory mediators. The elevation in the inflammatory response and oxidative stress may be important in AMS pathogenesis (Boos et al., 2016; Liu et al., 2017a). AA has been demonstrated to activate p38 mitogen-activated protein kinase (MAPK) and c-Jun N-terminal kinase (JNK), thereby directly promoting inflammation by increasing levels of tumor necrosis factor (TNF)-α (Saito et al., 2012). AA-derived metabolites such as leukotriene B4 (LTB4) and Cys–LTs from upregulated enzymes, including ALOX5 and GGT1, may operate as potent activators of and chemoattractants for leukocytes, eosinophils, and monocytes to propagate inflammation and oxidative stress (Rink and Khanna, 2011). Elevated PTGES may contribute to increased levels of PGE2 and PGA2, which activate IL-6 and nuclear factor kappa B (NF-κB) to promote vascular inflammation (Gomez et al., 2013). In addition to the biological effects of these metabolites, key enzymes in the AA metabolism pathway may directly trigger inflammation reactions and oxidative stress. Cyclooxygenase-2 (COX-2) contributes to the upregulation of TNF-α after hypoxia stimuli (Xing et al., 2015). The peroxidase activity of PTGS1 may serve as a source of oxygen radicals through the conversion of PGG2 to PGH2 by the removal of oxygen (Rink and Khanna, 2011). Moreover, phospholipase A2 may produce free radicals during the activation of arachidonic acid cascade under hypoxia environment (Tanaka et al., 2003). With limited evidence focusing on the role of the AA metabolism pathway in the development of hypoxic diseases, our results systematically reveal the expression level of the AA metabolism pathway as well as its possible roles based on the AMS model and provide the basis for further research.

The increase in metabolites indicates the upregulation in sub-pathways activated by COX, lipoxygenase, and PLA2—the key enzymes of the AA metabolism pathway (Wong et al., 2003; Jantan et al., 2014). In line with previous studies, these proteins showed a hypoxia-regulated pattern. A recent study demonstrated that hypoxia-mediated extracellular signal-regulated kinase (ERK) activation may induce the activity of PLA2 in the erythrocyte membrane (Wu et al., 2016). In addition, as an essential oxygen-sensing molecule, heme oxygenase-1 was reported to act as an important mediator of transcription and translation of 15-lipoxygenase to promote lipoxins (Nie et al., 2013). Hypoxia-inducible factor-1 alpha (HIF-1a) was reported to directly stimulate COX enzymes after hypoxia exposure (Huang et al., 2016). However, we failed to detect the quantitative expression of the key enzymes in the AA metabolism pathway, owing to the limited blood sample during validation. An intervention experiment with drugs targeting the AA metabolism pathway in larger population samples is now being conducted by our group to verify these findings and search for effective prevention measures.

In our study, we demonstrated a crosstalk between response manners to acute hypoxia and expression level of the AA metabolism pathway. The excessive increase in the AA metabolism pathway after hypoxia stimuli may reflect mal-adaptation to hypoxia stimuli, as evident from the elevated levels of HR, HB, and LLS as well as the decreased level of SpO_2_. These observations suggest impaired cardiopulmonary and hemoglobin function (Fuehrer and Huecker, 2017). For cardiopulmonary performance, the addition of AA was shown to aggravate respiration inhibition and decrease oxygen consumption at the mitochondrial level (Egorova et al., 2015), while targeting COX-2 improved hemodynamic parameters of cardiac output, left ventricular pressure, and LV*dp/dt* (Oshima et al., 2006). AA metabolism was also reported to influence heart rates by alerting histaminergic system (Altinbas et al., 2014). In addition, the elevated level of reactive oxygen species during AA metabolism was involved in the regulation of erythroid differentiation, and the activation of phospholipase A2 was shown to induce protein kinase C to increase erythrogenin level (Luo et al., 2017) (Mason-Garcia and Beckman, 1991). Furthermore, the process of reticulocyte maturation was dependent on phospholipase A2 during the remodeling of the plasma membrane by the removal of specific proteins (Blanc et al., 2007). As an irreversible pathological process with poor clinical outcomes caused by sustained hypoxia, elevated AA metabolism pathway may decrease the apoptosis of endothelial cells, induce the proliferation of pulmonary artery smooth muscle cells, activate angiogenesis, and influence the arterial vascular tonicity via K+ channels (Park et al., 2011; Zhang et al., 2011; Liu et al., 2012; Zhu and Ran, 2012; Pang et al., 2016). An excessive elevation in AA metabolism may result in poor response to acute hypoxia and even worse symptoms under chronic hypoxia environment.

The protective effects of anti-inflammatory molecules are increasingly recognized for the prevention of AMS. Apigenin targeting IL-1β, IL-6, and TNF-α was demonstrated to be effective in reducing the damage caused by acute hypoxia (Du et al., 2015). Recent pilot studies reported that ibuprofen, aspirin, and dexamethasone may be effective for the prevention and treatment of AMS, especially the syndrome of headache, although the underlying mechanisms remain largely unknown (Burtscher et al., 2001; Gertsch et al., 2012; Xiong et al., 2017). In this study, our results theoretically support anti-inflammatory drugs such as non-steroidal anti-inflammatory drugs (NSAIDS) and dexamethasone targeting the AA metabolism pathway for improving clinical symptoms of AMS. In addition, targeted therapies against specific enzymes or metabolites of the AA metabolism pathway may be beneficial in AMS prevention and demand further studies (Atluri et al., 2011). Given its close relationship with hemoglobin metabolism genes, the AA metabolism pathway may be an effective target for the prevention of hypoxia-induced erythrocytosis (Foley et al., 1978; Parise et al., 1991).

In summary, our integrated analysis of metabolomic and transcriptomic profiling revealed that the AA metabolism pathway was one of the most pivotal alterations after acute hypoxia exposure and may account for variations in response patterns to hypoxia stimuli. The detailed description of the AA metabolism pathway may offer the basis for follow-up mechanisms and drug screening.

## Availability of data

All data have been submitted to GEO under the accession GSE103940.

## Author contributions

YG and JC: Conceived and designed the study; CL and BL: Oversaw laboratory analyses and contributed the statistical analysis; LL, E-LZ, and BS: Contributed to sample and physical data collections; CL: Drafted the report. All authors reviewed and approved the manuscript.

### Conflict of interest statement

The authors declare that the research was conducted in the absence of any commercial or financial relationships that could be construed as a potential conflict of interest.

## References

[B1] Acharjee A. Kloosterman B. Visser R. G. Maliepaard C. (2016). Integration of multi-omics data for prediction of phenotypic traits using random forest. BMC Bioinformatics 17(Suppl. 5):180. 10.1186/s12859-016-1043-4 27295212 PMC4905610

[B2] Altinbas B. Topuz B. B. Ilhan T. Yilmaz M. S. Erdost H. Yalcin M. (2014). Activation of the central histaminergic system mediates arachidonic-acid-induced cardiovascular effects. Can. J. Physiol. Pharmacol. 92, 645–654. 10.1139/cjpp-2014-0043 25065747

[B3] Atluri D. K. Prakash R. Mullen K. D. (2011). Pathogenesis, diagnosis, and treatment of hepatic encephalopathy. J. Clin. Exp. Hepatol. 1, 77–86. 10.1016/S0973-6883(11)60126-6 25755319 PMC3940085

[B4] Bennett M. Gilroy D. W. (2016). Lipid mediators in inflammation. Microbiol. Spectr. 4. 10.1128/microbiolspec.MCHD-0035-2016 27837747

[B5] Blanc L. Barres C. Bette-Bobillo P. Vidal M. (2007). Reticulocyte-secreted exosomes bind natural IgM antibodies: involvement of a ROS-activatable endosomal phospholipase iPLA2. Blood 110, 3407–3416. 10.1182/blood-2007-04-085845 17666570

[B6] Bogatcheva N. V. Sergeeva M. G. Dudek S. M. Verin A. D. (2005). Arachidonic acid cascade in endothelial pathobiology. Microvasc. Res. 69, 107–127. 10.1016/j.mvr.2005.01.007 15896353

[B7] Boos C. J. Woods D. R. Varias A. Biscocho S. Heseltine P. Mellor A. J. (2016). high altitude and acute mountain sickness and changes in circulating Endothelin-1, Interleukin-6, and Interleukin-17a. High Alt. Med. Biol. 17, 25–31. 10.1089/ham.2015.0098 26680502

[B8] Burtscher M. Likar R. Nachbauer W. Philadelphy M. Pühringer R. Lämmle T. (2001). Effects of aspirin during exercise on the incidence of high-altitude headache: a randomized, double-blind, placebo-controlled trial. Headache 41, 542–545. 10.1046/j.1526-4610.2001.041006542.x 11437888

[B9] D'Alessandro A. Nemkov T. Sun K. Liu H. Song A. Monte A. A.. (2016). AltitudeOmics: red blood cell metabolic adaptation to high altitude hypoxia. J. Proteome Res. 15, 3883–3895. 10.1021/acs.jproteome.6b00733 27646145 PMC5512539

[B10] Du H. Hao J. Liu F. Lu J. Yang X. (2015). Apigenin attenuates acute myocardial infarction of rats via the inhibitions of matrix metalloprotease-9 and inflammatory reactions. Int. J. Clin. Exp. Med. 8, 8854–8859. 26309539 PMC4538101

[B11] Egorova M. V. Kutsykova T. V. Afanas'ev S. A. Popov S. V. (2015). Effect of arachidonic acid on the rate of oxygen consumption in isolated cardiomyocytes from intact rats and animals with ischemic or diabetic injury to the heart. Bull. Exp. Biol. Med. 160, 190–192. 10.1007/s10517-015-3124-1 26639470

[B12] Foley J. E. Gross D. M. Nelson P. K. Fisher J. W. (1978). The effects of arachidonic acid on erythropoietin production in exhypoxic polycythemic mice and the isolated perfused canine kidney. J. Pharmacol. Exp. Ther. 207, 402–409. 712628

[B13] Fuehrer J. Huecker M. R. (2017). Altitude Illness, High Altitude Cardiopulmonary Diseases. Treasure Island: FL: StatPearls. 28723040

[B14] Gertsch J. H. Corbett B. Holck P. S. Mulcahy A. Watts M. Stillwagon N. T.. (2012). Altitude sickness in climbers and efficacy of NSAIDs trial (ASCENT): randomized, controlled trial of ibuprofen versus placebo for prevention of altitude illness. Wilderness Environ. Med. 23, 307–315. 10.1016/j.wem.2012.08.001 23098412

[B15] Gomez I. Foudi N. Longrois D. Norel X. (2013). The role of prostaglandin E2 in human vascular inflammation. Prostaglandins Leukot. Essent. Fatty Acids 89, 55–63. 10.1016/j.plefa.2013.04.004 23756023

[B16] Gonggalanzi Labasangzhu Nafstad P. Stigum H. Wu T. Haldorsen Ø. D.. (2016). Acute mountain sickness among tourists visiting the high-altitude city of Lhasa at 3658 m above sea level: a cross-sectional study. Arch. Public Health 74:23. 10.1186/s13690-016-0134-z 27252854 PMC4888367

[B17] Huang M. Wang L. Chen J. Bai M. Zhou C. Liu S.. (2016). Regulation of COX-2 expression and epithelial-to-mesenchymal transition by hypoxia-inducible factor-1α is associated with poor prognosis in hepatocellular carcinoma patients post TACE surgery. Int. J. Oncol. 48, 2144–2154. 10.3892/ijo.2016.3421 26984380 PMC4809660

[B18] Jantan I. Bukhari S. N. Adekoya O. A. Sylte I. (2014). Studies of synthetic chalcone derivatives as potential inhibitors of secretory phospholipase A2, cyclooxygenases, lipoxygenase and pro-inflammatory cytokines. Drug Des. Devel. Ther. 8, 1405–1418. 10.2147/DDDT.S67370 25258510 PMC4172049

[B19] Johnson N. J. Luks A. M. (2016). High-altitude medicine. Med. Clin. North Am. 100, 357–369. 10.1016/j.mcna.2015.09.002 26900119

[B20] Kan M. Shumyatcher M. Himes B. E. (2017). Using omics approaches to understand pulmonary diseases. Respir. Res. 18:149. 10.1186/s12931-017-0631-9 28774304 PMC5543452

[B21] Li B. Bi C. L. Lang N. Li Y. Z. Xu C. Zhang Y. Q.. (2014). RNA-seq methods for identifying differentially expressed gene in human pancreatic islet cells treated with pro-inflammatory cytokines. Mol. Biol. Rep. 41, 1917–1925. 10.1007/s11033-013-3016-2 24619356

[B22] Li S. Todor A. Luo R. (2016). Blood transcriptomics and metabolomics for personalized medicine. Comput. Struct. Biotechnol. J. 14, 1–7. 10.1016/j.csbj.2015.10.005 26702339 PMC4669660

[B23] Liao W. T. Liu B. Chen J. Cui J. H. Gao Y. X. Liu F. Y.. (2016). Metabolite modulation in human plasma in the early phase of acclimatization to hypobaric hypoxia. Sci. Rep. 6:22589. 10.1038/srep22589 26940428 PMC4778071

[B24] Liu B. Chen J. Zhang L. Gao Y. Cui J. Zhang E.. (2017a). IL-10 dysregulation in acute mountain sickness revealed by transcriptome analysis. Front. Immunol. 8:628. 10.3389/fimmu.2017.00628 28611780 PMC5447681

[B25] Liu X. Hu A. X. Zhao J. L. Chen F. L. (2017b). Identification of key gene modules in human osteosarcoma by co-expression analysis weighted gene co-expression network analysis (WGCNA). J. Cell. Biochem. 118, 3953–3959. 10.1002/jcb.26050 28398605

[B26] Liu Y. Ma C. Zhang Q. Yu L. Ma J. Zhang L.. (2012). The key role of transforming growth factor-beta receptor I and 15-lipoxygenase in hypoxia-induced proliferation of pulmonary artery smooth muscle cells. Int. J. Biochem. Cell Biol. 44, 1184–1202. 10.1016/j.biocel.2012.04.009 22542888

[B27] Luo S. T. Zhang D. M. Qin Q. Lu L. Luo M. Guo F. C.. (2017). The promotion of erythropoiesis via the regulation of reactive oxygen species by lactic acid. Sci. Rep. 7:38105. 10.1038/srep38105 28165036 PMC5292721

[B28] Mason-Garcia M. Beckman B. S. (1991). Signal transduction in erythropoiesis. FASEB J. 5, 2958–2964. 10.1096/fasebj.5.14.1752362 1752362

[B29] Michiels C. (2004). Physiological and pathological responses to hypoxia. Am. J. Pathol. 164, 1875–1882. 10.1016/S0002-9440(10)63747-9 15161623 PMC1615763

[B30] Netzer N. C. Rausch L. Eliasson A. H. Gatterer H. Friess M. Burtscher M.. (2017). SpO2 and heart rate during a real hike at altitude are significantly different than at its simulation in normobaric hypoxia. Front. Physiol. 8:81. 10.3389/fphys.2017.00081 28243206 PMC5303738

[B31] Nie X. Hui Y. Shi S. Ma J. Wang S. Qiu Z.. (2013). Heme oxygenase-1 induces 15-lipoxygenase expression during hypoxia-induced pulmonary hypertension. Int. J. Biochem. Cell Biol. 45, 964–972. 10.1016/j.biocel.2013.01.017 23391748

[B32] Oshima K. Takeyoshi I. Tsutsumi H. Mohara J. Ohki S. Koike N.. (2006). Inhibition of cyclooxygenase-2 improves cardiac function following long-term preservation. J. Surg. Res. 135, 380–384. 10.1016/j.jss.2006.03.044 16713604

[B33] Pang L. Cai Y. Tang E. H. Yan D. Kosuru R. Li H.. (2016). Cox-2 inhibition protects against hypoxia/reoxygenation-induced cardiomyocyte apoptosis via Akt-dependent enhancement of iNOS expression. Oxid. Med. Cell. Longev. 2016:3453059. 10.1155/2016/3453059 27795807 PMC5067333

[B34] Parise P. Huybrechts E. Grasselli S. Falcinelli F. Nenci G. G. Gresele P.. (1991). Generation of arachidonic acid metabolites from stimulated whole blood in patients with chronic myeloproliferative disorders. Acta Haematol. 85, 88–92. 10.1159/000204863 1902615

[B35] Park S. J. Yoo H. Y. Earm Y. E. Kim S. J. Kim J. K. Kim S. D. (2011). Role of arachidonic acid-derived metabolites in the control of pulmonary arterial pressure and hypoxic pulmonary vasoconstriction in rats. Br. J. Anaesth. 106, 31–37. 10.1093/bja/aeq268 20935003

[B36] Rink C. Khanna S. (2011). Significance of brain tissue oxygenation and the arachidonic acid cascade in stroke. Antioxid. Redox Signal. 14, 1889–1903. 10.1089/ars.2010.3474 20673202 PMC3078506

[B37] Ritchie M. E. Phipson B. Wu D. Hu Y. Law C. W. Shi W.. (2015). Limma powers differential expression analyses for RNA-sequencing and microarray studies. Nucleic Acids Res. 43:e47. 10.1093/nar/gkv007 25605792 PMC4402510

[B38] Saito Y. Watanabe K. Fujioka D. Nakamura T. Obata J. E. Kawabata K.. (2012). Disruption of group IVA cytosolic phospholipase A(2) attenuates myocardial ischemia-reperfusion injury partly through inhibition of TNF-α-mediated pathway. Am. J. Physiol. Heart Circ. Physiol. 302, H2018–H2030. 10.1152/ajpheart.00955.2011 22427514 PMC3362116

[B39] Smoot M. E. Ono K. Ruscheinski J. Wang P. L. Ideker T. (2011). Cytoscape 2.8: new features for data integration and network visualization. Bioinformatics 27, 431–432. 10.1093/bioinformatics/btq675 21149340 PMC3031041

[B40] Subudhi A. W. Panerai R. B. Roach R. C. (2010). Effects of hypobaric hypoxia on cerebral autoregulation. Stroke 41, 641–646. 10.1161/STROKEAHA.109.574749 20185774

[B41] Sun K. Liu H. Song A. Manalo J. M. D'Alessandro A. Hansen K. C.. (2017). Erythrocyte purinergic signaling components underlie hypoxia adaptation. J. Appl. Physiol 123, 951–956. 10.1152/japplphysiol.00155.2017 28572494 PMC5668449

[B42] Sun Y. V. Hu Y. J. (2016). Integrative analysis of multi-omics data for discovery and functional studies of complex human diseases. Adv. Genet. 93, 147–190. 10.1016/bs.adgen.2015.11.004 26915271 PMC5742494

[B43] Tanaka E. Niiyama S. Sato S. Yamada A. Higashi H. (2003). Arachidonic acid metabolites contribute to the irreversible depolarization induced by *in vitro* ischemia. J. Neurophysiol. 90, 3213–3223. 10.1152/jn.00542.2003 12917387

[B44] Wong R. K. Pettit A. I. Quinn P. A. Jennings S. C. Davies J. E. Ng L. L. (2003). Advanced glycation end products stimulate an enhanced neutrophil respiratory burst mediated through the activation of cytosolic phospholipase A2 and generation of Arachidonic acid. Circulation 108, 1858–1864. 10.1161/01.CIR.0000089372.64585.3B 12963645

[B45] Wu H. Bogdanov M. Zhang Y. Sun K. Zhao S. Song A.. (2016). Hypoxia-mediated impaired erythrocyte lands' cycle is pathogenic for sickle cell disease. Sci. Rep. 6:29637. 10.1038/srep29637 27436223 PMC4951653

[B46] Xing Y. Wang R. Chen D. Mao J. Shi R. Wu Z.. (2015). COX2 is involved in hypoxia-induced TNF-α expression in osteoblast. Sci. Rep. 5:10020. 10.1038/srep10020 26066979 PMC4464352

[B47] Xiong J. Lu H. Wang R. Jia Z. (2017). Efficacy of ibuprofen on prevention of high altitude headache: a systematic review and meta-analysis. PLoS ONE 12:e0179788. 10.1371/journal.pone.0179788 28632763 PMC5478153

[B48] Yu G. Wang L. G. Han Y. He Q. Y. (2012). clusterProfiler: an R package for comparing biological themes among gene clusters. OMICS 16, 284–287. 10.1089/omi.2011.0118 22455463 PMC3339379

[B49] Zhang Q. Wang D. Singh N. K. Kundumani-Sridharan V. Gadiparthi L. Rao Ch M.. (2011). Activation of cytosolic phospholipase A2 downstream of the Src-phospholipase D1 (PLD1)-protein kinase C gamma (PKCgamma) signaling axis is required for hypoxia-induced pathological retinal angiogenesis. J. Biol. Chem. 286, 22489–22498. 10.1074/jbc.M110.217786 21536681 PMC3121394

[B50] Zhu D. Ran Y. (2012). Role of 15-lipoxygenase/15-hydroxyeicosatetraenoic acid in hypoxia-induced pulmonary hypertension. J. Physiol. Sci. 62, 163–172. 10.1007/s12576-012-0196-9 22331435 PMC10717549

