# Supplementary material for: Arachidonic Acid Metabolism Pathway Is Not Only Dominant in Metabolic Modulation but Associated With Phenotypic Variation After Acute Hypoxia Exposure
Source: Front Physiol. 2018 Mar 16;9:236. doi: 10.3389/fphys.2018.00236 (PMC5864929; doi:10.3389/fphys.2018.00236)
Supplement: Supplementary file 1 [file Table1.DOCX]

**Arachidonic acid metabolism pathway is not only dominant in metabolic modulation but associated with phenotypic variation after acute hypoxia exposure**

**Chang Liu^1,2,3^, Bao Liu^1,2,3^, Lu Liu^1,2,3^, Er-Long Zhang^1,2,3^, Binda Sun^1,2,3^, Gang Xu^1,2,3^, Chen Jian^1,2,3*^, Yuqi Gao^1,2,3*^**

**^*^Correspondence:**

Mr. Jian Chen or Yuqi Gao, E-mail: jchenone@163.com or gaoy66@yahoo.com

1. Supplementary Table

|  | Adaptation(N=12) | | maladaptation(N=14) | | p-value |
| --- | --- | --- | --- | --- | --- |
| SBP | 125.42(16.01) |  | 123.29(15.10) | | 0.73 |
| DBP | 70.58(11.70) |  | 69.79(13.47) | | 0.87 |
| HR | 86.67(15.22) |  | 102.43(14.31) | | 0.012 |
| SpO2 | 81.92(2.57) |  | 77.71(6.54) |  | 0.048 |
| LLS | 2.92(1.08) |  | 10.36(2.17) |  | 1.15E-10 |
| HB | 158.88(11.72) |  | 169.5(12.26) | | 0.034 |

Supplemental Table 1. Clinical features of enrolled subjects with diverse response patterns to hypoxia at high altitude.

1. Supplementary Figure


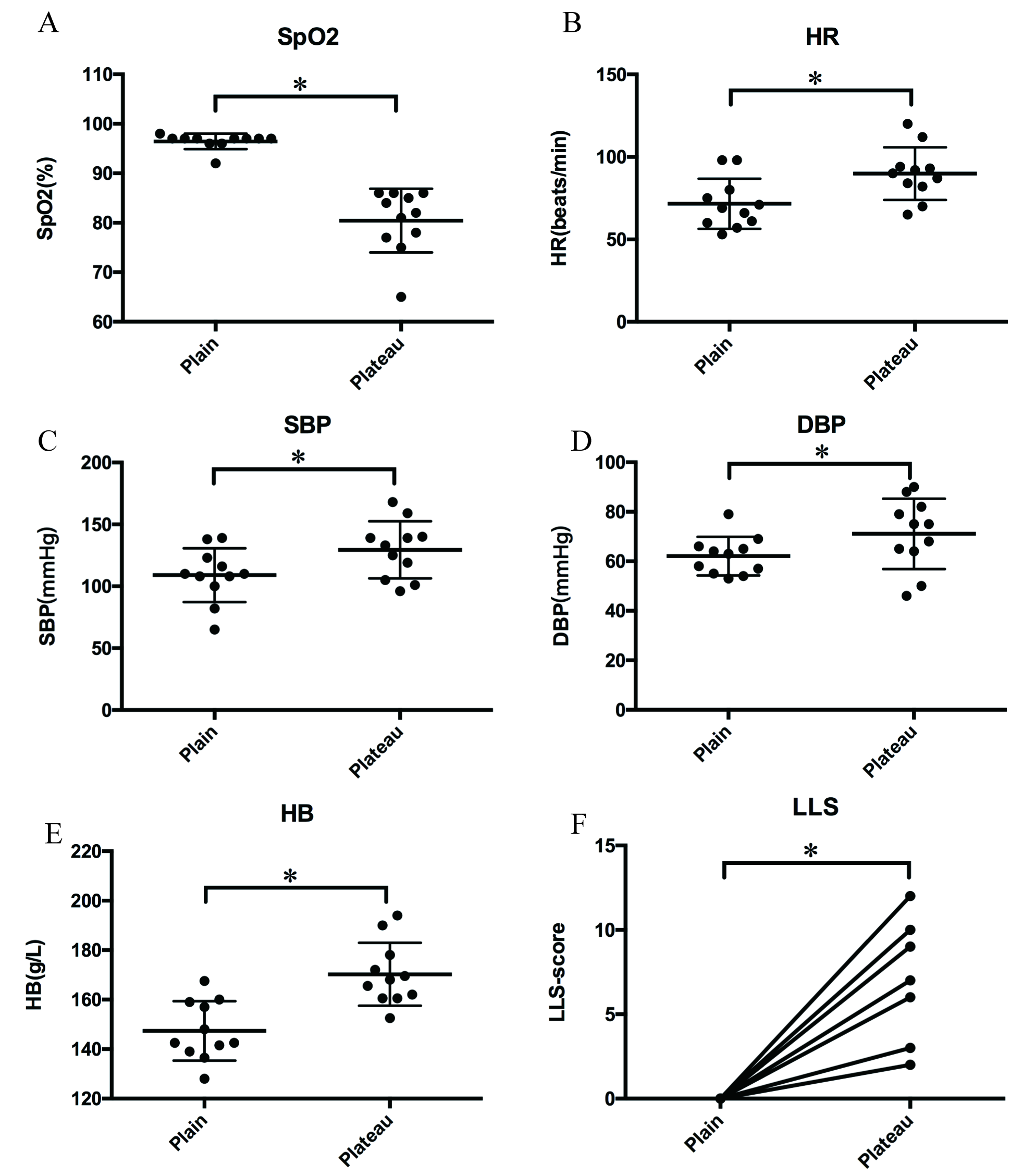


Supplementary Figure 1: The detailed clinic information of enrolled subjects in both metabolomics and transcriptomics profiling detection before and after hypoxia exposure.
